# Supplementary material for: Metabolic plasticity drives mismatches in physiological traits between prey and predator
Source: Commun Biol. 2024 May 28;7:653. doi: 10.1038/s42003-024-06350-y (PMC11133466; doi:10.1038/s42003-024-06350-y)
Supplement: Supplementary file 2 — Supplementary Information [file 42003_2024_6350_MOESM2_ESM.pdf]

Supplementary Information for

Metabolic plasticity drives mismatches in physiological traits between prey and predator

Flavio Affinito[1], Miguel Matias[2], Rebecca L. Kordas[1] and Samraat Pawar[1]

1. Department of Life Sciences, Imperial College London, Silwood Park Buckhurst Road, SL5 7PY, Ascot UK

2. Museo Nacional de Ciencias Naturales (CSIC), Madrid, 28006, Spain

Corresponding author: *E-mail: flavio.affinito@mail.mcgill.ca*

SUPLEMENTARY METHODS 1

Feeding trials

Supplementary Table 1 Length-weight regression equations, where *L* stands for length.

SUPPLEMENTARY METHODS 2

Velocity estimation

Search rate model derivation

Dimensionality effect

Biological rates and temperature-dependence

Supplementary Table 2. Sharpe-Schoolfield model parameters and fit.
Supplementary Table 3. Activation energies of predator search rates.
Supplementary Figure 1. The Iberian Ponds network.
Supplementary Figure 2. Yearly recorded temperatures at each site.
Supplementary Figure 3. Mean monthly temperatures recorded during the larval stage of all taxa.

SUPPLEMENTARY REFERENCES

SUPPLEMENTARY METHODS 1

Feeding trials

To determine predator-prey relationships between species, we conducted feeding trials at each site. For each potential predator-prey pair, we placed one individual of a predator species into a water filled jar (50 ml), then added 2 individuals of a potential prey species. Individuals were left in jars overnight and the number of prey individuals was counted in the morning. These trials revealed a predatory relationship between the dragonfly species *Sympetrum striolatum* and two prey taxa, the mayfly species *Cloeon dipterum* and the chironomid genus *Chironomus*. Some taxa were not found in high enough abundance at some sites to use in respirometry experiments due to differences in larval phenology (Supplementary Figure 1).

Supplementary Table 1 **Length-weight regression equations, where *L* stands for length.** Individuals were measured under the microscope and placed in an individual foil cup. *Cloeon* and *Sympetrum* individuals were measured from the tip of the abdomen to the midpoint between the eyes and all antennae were ignored. *Chironomus* individuals were measured from the mouth to the base of the terminal prolegs. All cups were labelled and left in an oven at 80°C for 16 to 18 hours to dry completely. Dry-weights were then recorded for each individual. *Cloeon* and *Sympetrum* linear models yielded satisfactory fit (R^2 > 0.6), *Chironomidae* was taken from the literature.

Taxa	Regression	R^2	N	Reference
Odonata	$W = -0.63.10^{-3} + 0.22.10^{-3}L$	0.88	49	
Ephemeroptera	$W = e^{-10.12+0.41L}$	0.65	104	
Chironomidae	$W = 0.6.10^{-3}L^{2.77}$	0.86		Benke *et al.*, 1999

SUPPLEMENTARY METHODS 2

Velocity calculation

The taxa used in this study are swimmers. The energetics of swimming have been studied for various species (Videler and Nolet, 1990; Videler, 1993; Alexander, 2003). Assuming velocity scales linearly with metabolic rate (Tucker, 1970), we can convert a measure of oxygen consumption (B; equation 1) into one of velocity. The relationship between respiration and velocity is linked to a measure of the cost of transport (C): the amount of energy in J needed to transport 1N over 1m in submerged swimmers (Videler, 1993). Thus, we express velocity as:

$$v = \frac{B_j}{Cmg} \quad \text{(S1)}$$

Where B_j is oxygen consumption converted in J.s^{-1}, C is in J.N^{-1}.m^{-1}, m is mean mass in kg

and g is gravitational acceleration in m.s^{-2}. Cost of transport in swimmers scales with body mass as follows (Videler and Nolet, 1990):

$$C = 1.1m^{-0.038} \quad \text{(S2)}$$

Oxygen consumption for nutrient combustion is the primary means by which nutrients are converted into energy. Oxygen consumption, measured in μmol.h^{-1} can be converted to g.h^{-1} by multiplying by the atomic mass of O_2: 31.988g/mol. The combustion of carbohydrates, fat and protein yields 3.34cal per 1mg of oxygen (Elliott and Davison, 1975) and 1cal yields 4.2868J (Merrill and Watt, 1973; Food and Agriculture Organization, 2015). Thus, we estimate the energetic output of respiration by defining a conversion coefficient of oxygen combustion:

$$\gamma = M_{O_2} K E_k \quad \text{(S3)}$$

Where M_{O2} is the atomic mass of oxygen, K is the caloric value of oxygen combustion and E_k is the energetic value of a calorie. We can express velocity's temperature dependence with respect to metabolic rate as:

$$v = \frac{\left(\frac{B_0 m^{\beta} e^{\frac{-E}{k}\left(\frac{1}{T} - \frac{1}{T_{ref}}\right)}}{1 + e^{\frac{E_d}{k}\left(\frac{1}{T_{pk}} - \frac{1}{T}\right)}} \right) \gamma}{1.1 m^{0.962} g} \quad \text{(S4)}$$

Search rate model

Dimensionality effect

The dimensionality component D for equation 4 expands into (Pawar, Dell and Savage, 2012):

$$D = d^{(D_m - 1)} s_D \quad \text{(S5)}$$

Species interactions in nature can be defined as either 2D (D_m=2; s_D=2) or 3D (D_m=3; s_D=π) dependent on the environment they interact in (Pawar, Dell and Savage, 2012). In this component, d is the detection distance of a predator and is dependent upon the respective body masses of the predator-prey pair:

$$d = d_0 (m_r m_c)^{p_d} \quad \text{(S6)}$$

Where d_0 is the minimum detection distance, m_r and m_c are prey and predator mass respectively and p_d is the empirical scaling exponent of dimensionality(Pawar, Dell and Savage, 2012). Thus, when considering the effect of dimensionality on search rates, we obtain two scenarios:

$$2D: D = 2d_0(m_r m_c)^{p_d} \tag{S7}$$

$$3D: D = \pi(d_0(m_r m_c)^{p_d})^2 \tag{S8}$$

Biological rates and temperature-dependence

Relative body velocity is expressed as (Dell, Pawar and Savage, 2011, 2014):

$$v = \sqrt{{v_r}^2 + {v_c}^2} \tag{S9}$$

Where v_r and v_c are prey and predator body velocities respectively. Here, we consider two interaction cases, one where prey species are relatively sessile compared to the predator ($v_r \approx v_c$) and one where both predator and prey are in active movement and equation S4 holds true. Predator and prey velocities as a biological rates scale with temperature and mass:

$$v_{c,r} = v_{0_{c,r}} {m_{c,r}}^{\beta_{c,r}} e^{\frac{-E_{c,r}}{k}\left(\frac{1}{T} - \frac{1}{T_{ref}}\right)} \tag{S10}$$

Where c and r subscripts apply to predator(consumer) and prey (resource) respectively, v_0 is the baseline trait performance at a reference temperature (T_{ref}), m is mass, β is the mass scaling exponent, E is activation energy and T is temperature. Hence when the prey is considered sessile we get:

$$2D: a = v_{0_c} {m_c}^{\beta_c} e^{\frac{-E_c}{k}\left(\frac{1}{T} - \frac{1}{T_{ref}}\right)} 2d_0(m_r m_c)^{p_d} \tag{S11}$$

$$3D: a = v_{0_c} {m_c}^{\beta_c} e^{\frac{-E_c}{k}\left(\frac{1}{T} - \frac{1}{T_{ref}}\right)} \pi(d_0(m_r m_c)^{p_d})^2 \tag{S12}$$

When both predator and prey species are active we have:

$$v_r = \sqrt{{v_{0_r}}^2 {m_r}^{2\beta_r} e^{\frac{-2E_r}{k}\left(\frac{1}{T} - \frac{1}{T_{ref}}\right)} + {v_{0_c}}^2 {m_c}^{2\beta_c} e^{\frac{-2E_c}{k}\left(\frac{1}{T} - \frac{1}{T_{ref}}\right)}} \tag{S13}$$

Thus, for active predator-prey search rate models we have:

$$2D: a = \sqrt{{v_{0_r}}^2 {m_r}^{2\beta_r} e^{\frac{-2E_r}{k}\left(\frac{1}{T} - \frac{1}{T_{ref}}\right)} + {v_{0_c}}^2 {m_c}^{2\beta_c} e^{\frac{-2E_c}{k}\left(\frac{1}{T} - \frac{1}{T_{ref}}\right)}} . 2d_0(m_r m_c)^{p_d} \tag{S14}$$

$$3D: a = \sqrt{{v_{0_r}}^2 {m_r}^{2\beta_r} e^{\frac{-2E_r}{k}\left(\frac{1}{T} - \frac{1}{T_{ref}}\right)} + {v_{0_c}}^2 {m_c}^{2\beta_c} e^{\frac{-2E_c}{k}\left(\frac{1}{T} - \frac{1}{T_{ref}}\right)}} . \pi(d_0(m_r m_c)^{p_d})^2 \tag{S15}$$

Supplementary Table 2 **Parameter estimates and model fits for all Sharpe-Schoolfield models.**

Species	Site	Parame-ter	Value	SE	t value	p value	R^2	N

Chironomus spp.	Toledo	B_0	0.198	0.065	3.05	0.003	0.56	108
		E	0.533	0.076	7.0	<0.001		
		β	1.38	0.262	5.23	<0.001		
		E_d	32.4	$2 .10^{7}$	$1.73 .10^{-6}$	1		
		T_{pk}	316.7	$4 .10^{5}$	$8.4 .10^{-4}$	1		
Chironomus spp.	Porto	B_0	0.107	0.026	4.05	<0.001	0.79	110
		E	0.614	0.040	15.4	<0.001		
		β	0.910	0.257	3.54	<0.001		
		E_d	32.1	$1 .10^{7}$	$2.96 .10^{-6}$	1		
		T_{pk}	316.8	$4 .10^{5}$	$8.8 .10^{-4}$	1		
Chironomus spp.	Evora	B_0	0.102	0.059	1.72	0.09	0.68	164
		E	0.598	0.042	14.1	<0.001		
		β	0.943	0.518	1.82	0.07		
		E_d	32.1	$1 .10^{7}$	$3.05 .10^{-6}$	1		
		T_{pk}	316.7	$7 .10^{5}$	$4.6 .10^{-4}$	1		
Cloeon dipterum	Toledo	B_0	0.046	0.009	5.12	<0.001	0.82	86
		E	1.68	0.203	8.27	<0.001		
		β	0.5	0.353	1.42	0.16		
		E_d	2.09	0.284	7.36	<0.001		
		T_{pk}	310.0	5.06	151	<0.001		
Cloeon dipterum	Porto	B_0	0.184	0.042	4.38	<0.001	0.79	102
		E	0.770	0.062	12.4	<0.001		
		β	0.5	0.580	0.86	0.39		
		E_d	2.95	1.09	2.70	0.008		
		T_{pk}	311.1	1.02	306	<0.001		
Cloeon dipterum	Evora	B_0	0.245	0.035	6.99	<0.001	0.72	109
		E	0.665	0.057	11.7	<0.001		
		β	0.617	0.295	2.09	0.04		
		E_d	4.18	1.50	2.79	0.006		
		T_{pk}	312.1	1.54	203	<0.001		
Sympetrum striolatum	Toledo	B_0	0.050	0.037	1.35	0.18	0.57	75
		E	1.96	0.554	3.53	<0.001		
		β	0.5	1.01	0.46	0.62		
		E_d	2.02	0.447	4.52	<0.001		
		T_{pk}	310.3	12.3	23.4	<0.001		
Sympetrum striolatum	Porto	B_0	0.152	0.014	10.6	<0.001	0.69	94
		E	0.773	0.075	10.3	<0.001		
		β	1.69	0.39	4.30	<0.001		
		E_d	7.48	2.77	2.70	0.008		
		T_{pk}	312.5	1.57	199	<0.001		
Sympetrum striolatum	Evora	B_0	0.246	0.093	2.64	0.009	0.59	111
		E	0.802	0.096	8.37	0.04		
		β	1.24	0.589	2.11	<0.001		
		E_d	2.56	0.448	5.72	<0.001		
		T_{pk}	307.5	1.19	258	<0.001		

Supplementary Table 3. **Activation energies of predator search rates.**

Prey	Site	Strategy	Activation energy (E)	SE	p value
Chironomus spp.	Toledo	Sessile 2D	1.96	$2.55 .10^{-16}$	<0.001
Chironomus spp.	Porto	Sessile 2D	0.77	$1.79 .10^{-16}$	<0.001
Chironomus spp.	Evora	Sessile 2D	0.80	$1.58 .10^{-16}$	<0.001
Cloeon dipterum	Toledo	Active 3D	1.85	$1.88 .10^{-3}$	<0.001
Cloeon dipterum	Porto	Active 3D	0.77	$1.43 .10^{-7}$	<0.001
Cloeon dipterum	Evora	Active 3D	0.74	$5.04 .10^{-4}$	<0.001

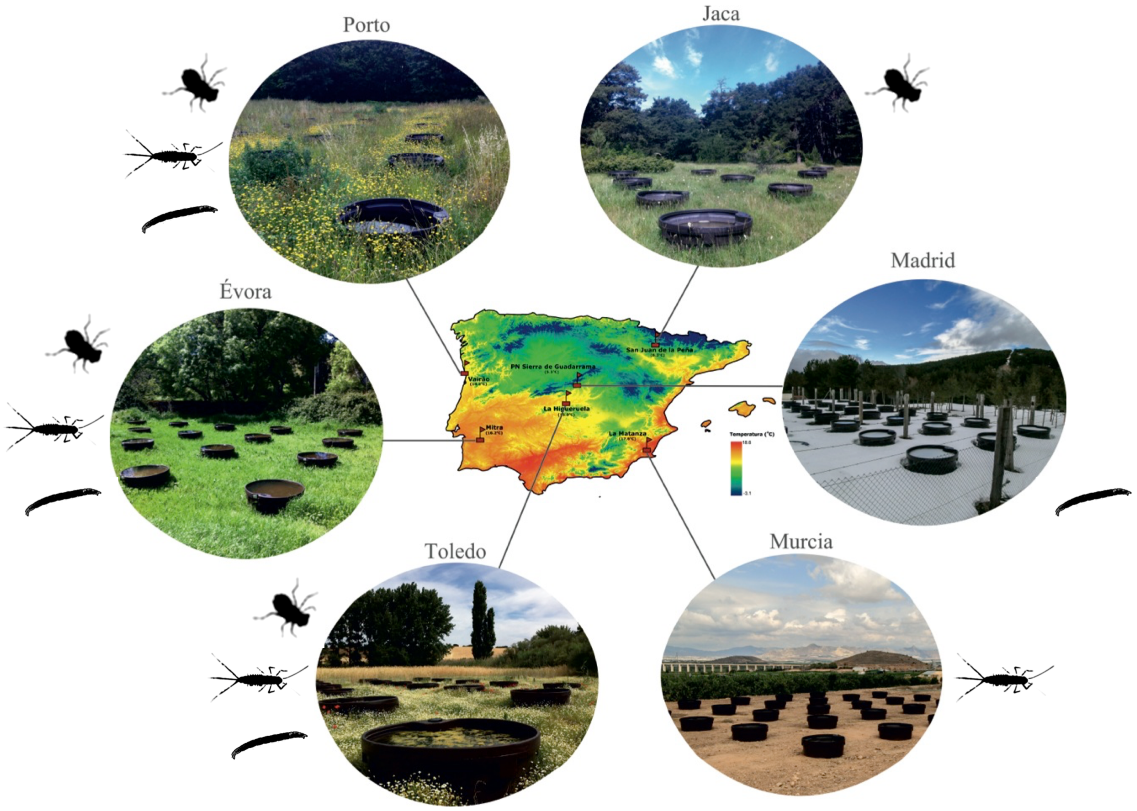

Supplementary Figure 1. **The Iberian Ponds network is located in the thermally diverse Iberian Peninsula.** The location of all six mesocosm experimental sites is shown with respect to a thermal map of the peninsula. Individual taxa sampled at each site are displayed (Jaca: *S. striolatum* alone; Madrid: *Chironomus* spp. alone; Murcia: *C. cloeon* alone). Figure was created by Miguel Matias with photos taken by Miguel Matias. This figure is also used in other material on the Iberian Ponds Network.

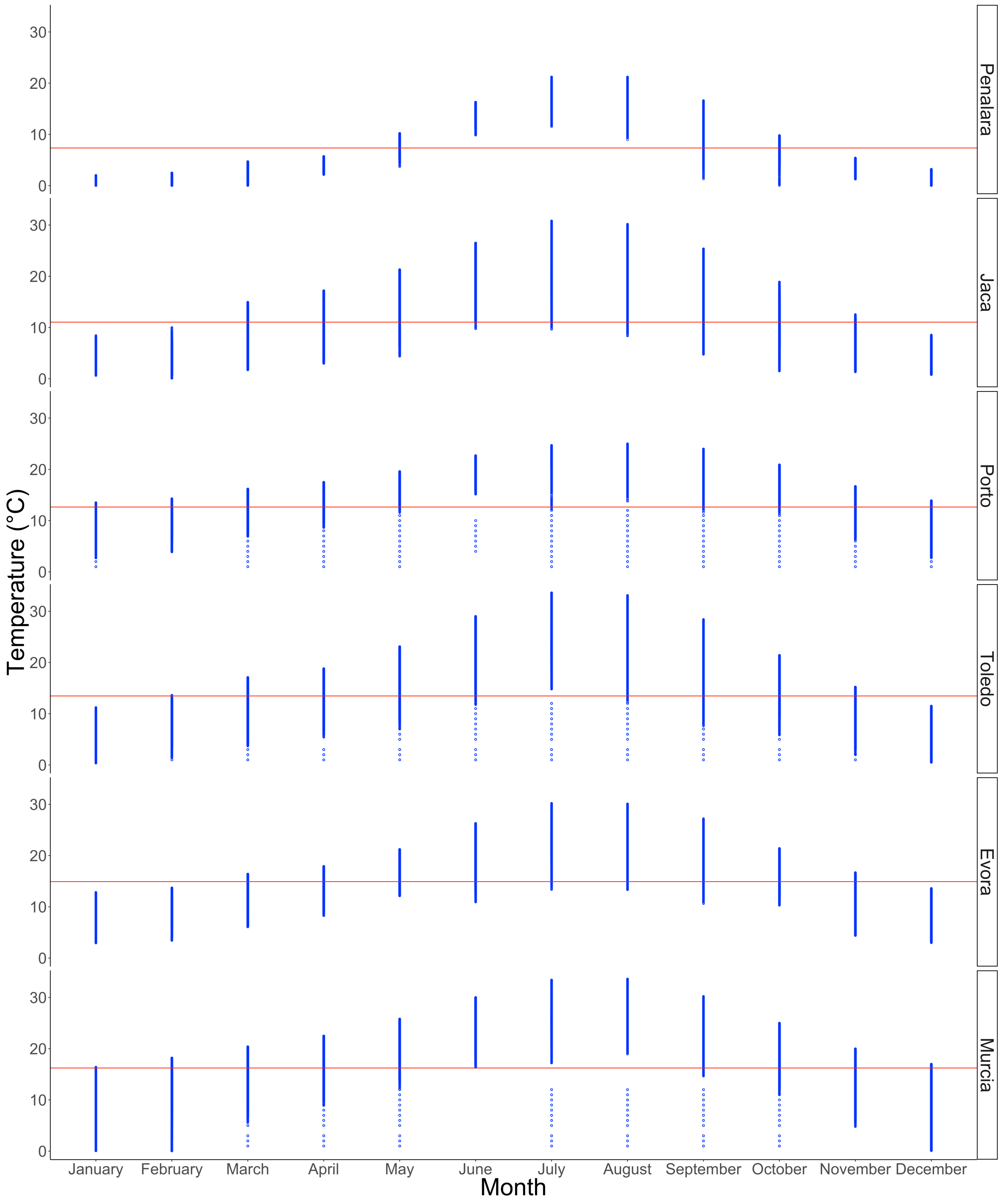

Supplementary Figure 2 **Yearly recorded temperatures at each site.** Temperatures recorded by loggers over 2-3 years plotted per month. Site mean temperature added in red.

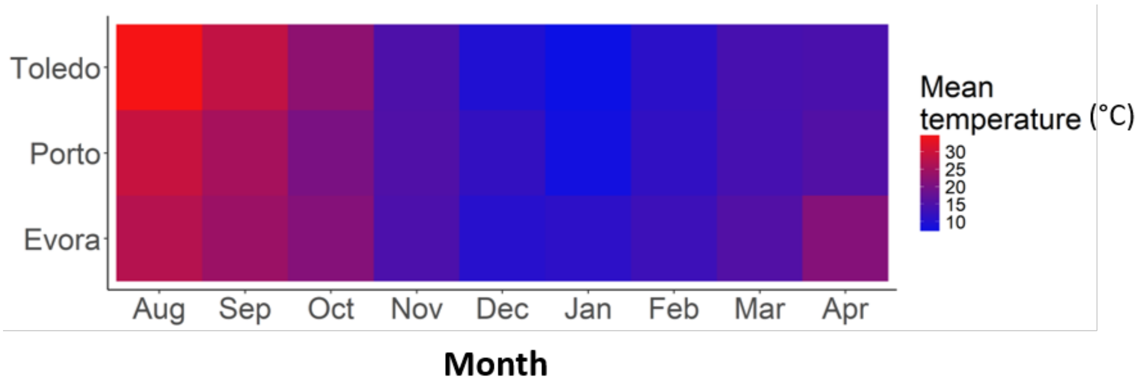

Supplementary Figure 3. **Mean monthly water temperatures recorded at each site in the Iberian Ponds network, during the aquatic stage of the individuals used in our experiments (August 2016 to April 2017).**

SUPPLEMENTARY REFERENCES

Alexander, R.M. (2003) *Principles of animal locomotion*. Princeton University Press.

Benke, A.C., Huryn, A.D., Smock, L.A. and Wallace, J.B., 1999. Length-mass relationships for freshwater macroinvertebrates in North America with particular reference to the southeastern United States. *Journal of the North American Benthological Society*, *18*(3), pp.308-343.

Dell, A.I., Pawar, S. and Savage, V.M. (2011) 'Systematic variation in the temperature dependence of physiological and ecological traits', *Proceedings of the National Academy of Sciences*, 108(26), pp. 10591–10596. Available at: https://doi.org/10.1073/pnas.1015178108.

Dell, A.I., Pawar, S. and Savage, V.M. (2014) 'Temperature dependence of trophic interactions are driven by asymmetry of species responses and foraging strategy', *Journal of Animal Ecology*, 83(1), pp. 70–84. Available at: https://doi.org/10.1111/1365-2656.12081.

Elliott, J.M. and Davison, W. (1975) 'Energy equivalents of oxygen consumption in animal energetics', *Oecologia*, 19(3), pp. 195–201. Available at: https://doi.org/10.1007/BF00345305.

Food and Agriculture Organization (2015) *FAOSTAT*, *United Nations*.

Merrill, A.L. and Watt, B.K. (1955) *Energy Value of Foods: Basis and Derivation,*. Human Nutrition Research Branch, Agricultural Research Service, U. S. Department of Agriculture.

Pawar, S., Dell, A.I. and Savage, V.M. (2012) 'Dimensionality of consumer search space drives trophic interaction strengths', *Nature*, 486(7404), pp. 485–489. Available at: https://doi.org/10.1038/nature11131.

Tucker, V.A. (1970) 'Energetic cost of locomotion in animals', *Comparative Biochemistry and Physiology*, 34(4), pp. 841–846.

Tucker, V.A. and Catlett, R. (1973) 'Energetic Cost of Locomotion in Animals', in *Readings in Animal Energetics*. Ardent Media, pp. 34–109.

Videler, J.J. and Nolet, B.A. (1990) 'Costs of swimming measured at optimum speed: scale effects, differences between swimming styles, taxonomic groups and submerged and surface swimming', *Comparative Biochemistry and Physiology Part A: Physiology*, 97(2), pp. 91–99.

Videler, J.J. (1993) *Fish swimming*. Springer Science & Business Media.
