# Supplementary material for: Metabolic plasticity drives mismatches in physiological traits between prey and predator
Source: Commun Biol. 2024 May 28;7:653. doi: 10.1038/s42003-024-06350-y (PMC11133466; doi:10.1038/s42003-024-06350-y)
Supplement: Supplementary file 3 — Reporting Summary [file 42003_2024_6350_MOESM3_ESM.pdf]

nature portfolio

Corresponding author(s): Flavio Affinito

Last updated by author(s): Nov 15, 2023

Reporting Summary

Nature Portfolio wishes to improve the reproducibility of the work that we publish. This form provides structure for consistency and transparency in reporting. For further information on Nature Portfolio policies, see our Editorial Policies and the Editorial Policy Checklist.

Statistics

For all statistical analyses, confirm that the following items are present in the figure legend, table legend, main text, or Methods section.

n/a	Confirmed	
☐	☒	The exact sample size (*n*) for each experimental group/condition, given as a discrete number and unit of measurement
☐	☒	A statement on whether measurements were taken from distinct samples or whether the same sample was measured repeatedly
☐	☒	The statistical test(s) used AND whether they are one- or two-sided<br>*Only common tests should be described solely by name; describe more complex techniques in the Methods section.*
☐	☒	A description of all covariates tested
☐	☒	A description of any assumptions or corrections, such as tests of normality and adjustment for multiple comparisons
☐	☒	A full description of the statistical parameters including central tendency (e.g. means) or other basic estimates (e.g. regression coefficient) AND variation (e.g. standard deviation) or associated estimates of uncertainty (e.g. confidence intervals)
☒	☐	For null hypothesis testing, the test statistic (e.g. *F*, *t*, *r*) with confidence intervals, effect sizes, degrees of freedom and *P* value noted<br>*Give P values as exact values whenever suitable.*
☒	☐	For Bayesian analysis, information on the choice of priors and Markov chain Monte Carlo settings
☒	☐	For hierarchical and complex designs, identification of the appropriate level for tests and full reporting of outcomes
☒	☐	Estimates of effect sizes (e.g. Cohen's *d*, Pearson's *r*), indicating how they were calculated

Our web collection on statistics for biologists contains articles on many of the points above.

Software and code

Policy information about availability of computer code

Data collection	Oxygen was continuously monitored using an oxygen microelectrode (MicroResp EL; Unisense, Denmark) and the signal from the multi-channel pA meter (PA8000; Unisense) was logged every 10s using an a/d converter (ADC16; Pico Technology, St Neots, U.K.) connected to a computer. All data were collected directly in the field and entered into Excell files. Data were then processed in R using R Studio.
Data analysis	Only R and R Studio were used for analysis.

For manuscripts utilizing custom algorithms or software that are central to the research but not yet described in published literature, software must be made available to editors and reviewers. We strongly encourage code deposition in a community repository (e.g. GitHub). See the Nature Portfolio guidelines for submitting code & software for further information.

Data

Policy information about availability of data

All manuscripts must include a data availability statement. This statement should provide the following information, where applicable:

- Accession codes, unique identifiers, or web links for publicly available datasets
- A description of any restrictions on data availability
- For clinical datasets or third party data, please ensure that the statement adheres to our policy

Data used to produce this manuscript can be accessed freely at: https://github.com/FlavAff/Locomotion-metabolism-and-acclimation

Research involving human participants, their data, or biological material

Policy information about studies with human participants or human data. See also policy information about sex, gender (identity/presentation), and sexual orientation and race, ethnicity and racism.

Reporting on sex and gender	N/A
Reporting on race, ethnicity, or other socially relevant groupings	N/A
Population characteristics	N/A
Recruitment	N/A
Ethics oversight	N/A

Note that full information on the approval of the study protocol must also be provided in the manuscript.

Field-specific reporting

Please select the one below that is the best fit for your research. If you are not sure, read the appropriate sections before making your selection.

☐ Life sciences ☐ Behavioural & social sciences ☒ Ecological, evolutionary & environmental sciences

For a reference copy of the document with all sections, see nature.com/documents/nr-reporting-summary-flat.pdf

Ecological, evolutionary & environmental sciences study design

All studies must disclose on these points even when the disclosure is negative.

Study description	The study analysed respiration rates of invertebrate taxa present in a series of experimental ponds set up across the Iberian peninsula along a natural temperature gradient. Communities were left to assemble naturally over two years prior to the study period. Organisms were then collected from ponds at each site depending on abundance. Each site had 32 ponds but not all ponds had equal abundances of invertebrates. We collected organisms from the ponds with highest abundances at each site. These organisms were then taken to the lab, where their respiration rates were measured in individual chambers.
Research sample	We first identified which macroinvertebrates were most abundant at each site and focused on sampling and measuring respiration rates for these. We found that organisms from Odonata, Ephemeroptera and Chironomidae were very abundant and therefore focused on those. We also noted a predatory relationship between Odonata and the other two taxa. We focused on these taxa as they were present and abundant at all experimental sites.
Sampling strategy	Sample sizes were dependent on the abundance of macroinvertebrates present at each sampling location. Experimental setups allowed for up to 7 specimens to be tested at a time for each temperature setting where enough were available. Total sample size was 959 across all sites and all species. Details of sample sizes per species and site are found in the supplementary material. These sample sizes were deemed sufficient for analysis based on previously published research on metabolic measurements of invertebrates by co-authors of this article and others in the field. Site specific ample sizes varied from a low of 75 to a high of 164 due to natural abundance at each site.
Data collection	Data were collected by placing each of the collected individuals in independent chambers. Oxygen was continuously monitored using an oxygen microelectrode (MicroResp EL; Unisense, Denmark) and the signal from the multi-channel pA meter (PA8000; Unisense) was logged every 10s using an a/d converter (ADC16; Pico Technology, St Neots, U.K.) connected to a computer. These trials were repeated for each temperature 7 times. These measurements were done by Flavio Affinito (FA) & Rebecca Kordas (RK). Collection of samples was done by FA, RK and Miguel Matias.
Timing and spatial scale	All the data used in this article were collected in March 2017. We spent a total of 5 days at each site (Toledo, Evora then Porto) to collect organisms and run experiments. At each site, the first day was used to collect organisms and the following days for respiration measurements. If too few organisms were collected to have 7 repeats per temperature, other organisms were additionally collected after day 1.
Data exclusions	Data from oxygen sensors was checked during each trial for manipulation errors from a misplaced sensor and notes were written for samples where this was the case. Samples with clear manipulation errors were excluded. One sample was excluded because an air bubble was found in the tube, another because the individual began pupating during the experiment. These were due to the samples violating inclusion criteria.
Reproducibility	All measurements were replicated a minimum of 7 times. Where more individuals were present and time allowed replications went up to 14 times per temperature x species. The experimental procedure followed pre-established and fully reproducible methodology.
Randomization	Organisms were collected randomly and starved in tubs by species. Individuals were then selected at random for each trial, data

Randomization	were corrected by mass in all cases to account for differences in body size.
Blinding	Blinding was not deemed relevant, organisms could not be distinguished to the species level during experimental handling.

Did the study involve field work? ☒ Yes ☐ No

Field work, collection and transport

Field conditions	Field season took place in the spring in warm sunny conditions at all sites.
Location	Sampling sites were on the grounds of the university of Evora, by the national research centres in Murcia and Porto.
Access & import/export	All materials were acquired in Madrid by via providers of the Natural History Museum, no permits or paperwork was required to transport them between EU countries.
Disturbance	The study took place in an experimental set up with no disturbance to the surroundings.

Reporting for specific materials, systems and methods

We require information from authors about some types of materials, experimental systems and methods used in many studies. Here, indicate whether each material, system or method listed is relevant to your study. If you are not sure if a list item applies to your research, read the appropriate section before selecting a response.

Materials & experimental systems

n/a	Involved in the study	
☒	☐	Antibodies
☒	☐	Eukaryotic cell lines
☒	☐	Palaeontology and archaeology
☐	☒	Animals and other organisms
☒	☐	Clinical data
☒	☐	Dual use research of concern
☒	☐	Plants

Methods

n/a	Involved in the study	
☒	☐	ChIP-seq
☒	☐	Flow cytometry
☒	☐	MRI-based neuroimaging

Animals and other research organisms

Policy information about studies involving animals; ARRIVE guidelines recommended for reporting animal research, and Sex and Gender in Research

Laboratory animals	The study did not involve laboratory animals.
Wild animals	Invertebrates were collected from mesocosms with nets and placed into large water containers to be taken to the lab. Ondonata were separated from Ephemeroptera and Chirnomidae (species level identification was not possible at this point) to avoid predation and allow time for gut clearing. All individuals were then handled with pipettes or tweezers when pipettes were found to be too small. After experiments, organisms were placed in ethanol filled eppendorf tubes for transport back to the Natural History Museum where they could be identified to the species level. Identification require microscopy and, in the case of Chironomidae, sample destruction which required killing the organisms.
Reporting on sex	Sex based analysis was not thought to be relevant and identification of sex was not feasible for these taxa.
Field-collected samples	After sampling from mesocosms, organisms were held in large plastic containers of mesocosm water held at ambient temperature over night. After experimental trials were conducted, organisms were placed in labeled ethanol filled eppendorf tubes for transport.
Ethics oversight	No ethical approval was required for working with insects.

Note that full information on the approval of the study protocol must also be provided in the manuscript.

Plants

Seed stocks	N/A
Novel plant genotypes	N/A
Authentication	N/A
